# High Spatiotemporal Resolution Radial Encoding Single‐Vessel fMRI

**DOI:** 10.1002/advs.202309218

**Published:** 2024-04-30

**Authors:** Yuanyuan Jiang, Patricia Pais‐Roldán, Rolf Pohmann, Xin Yu

**Affiliations:** ^1^ Athinoula A. Martinos Center for Biomedical Imaging Massachusetts General Hospital and Harvard Medical School Charlestown MA 02129 USA; ^2^ Institute of Neuroscience and Medicine 4 Medical Imaging Physics Forschungszentrum Jülich 52425 Jülich Germany; ^3^ High‐Field Magnetic Resonance Max Planck Institute for Biological Cybernetics 72076 Tübingen Germany

**Keywords:** high spatiotemporal resolution, radial encoding, single‐vessel fMRI

## Abstract

High‐field preclinical functional MRI (fMRI) is enabled the high spatial resolution mapping of vessel‐specific hemodynamic responses, that is single‐vessel fMRI. In contrast to investigating the neuronal sources of the fMRI signal, single‐vessel fMRI focuses on elucidating its vascular origin, which can be readily implemented to identify vascular changes relevant to vascular dementia or cognitive impairment. However, the limited spatial and temporal resolution of fMRI is hindered hemodynamic mapping of intracortical microvessels. Here, the radial encoding MRI scheme is implemented to measure BOLD signals of individual vessels penetrating the rat somatosensory cortex. Radial encoding MRI is employed to map cortical activation with a focal field of view (FOV), allowing vessel‐specific functional mapping with 50 × 50 µm^2^ in‐plane resolution at a 1 to 2 Hz sampling rate. Besides detecting refined hemodynamic responses of intracortical micro‐venules, the radial encoding‐based single‐vessel fMRI enables the distinction of fMRI signals from vessel and peri‐vessel voxels due to the different contribution of intravascular and extravascular effects.

## Graphical abstract

Herein, radial encoding single‐vessel fMRI is applied to map cortical activation, showcasing a unique field of view flexibility (1‐2 Hz sampling rate) and real‐time fMRI with a remarkable 50 x 50 µm^2^ in‐plane resolution. Notably, the high‐resolution fMRI methodology enables differentiate between fMRI signals from vessels and peri‐vessel voxels, owing to the distinct contributions of intravascular and extravascular effects.

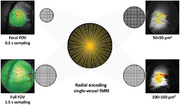

## 1 Introduction

Conventional functional magnetic resonance imaging (fMRI) methods^[^ ^1^, ^3^, ^4^ ^]^ are developed to measure the hemodynamic response as a surrogate of neuronal activity. The vascular origin of fMRI signals can be specified as changes in blood volume, flow, and oxygenation saturation driven by neurovascular coupling. Since the exact volume contribution of the cerebrovascular to the brain is less than ≈2–4%,^[^ ^5^, ^6^, ^7^ ^]^ fMRI signals of a given voxel with sub‐millimeter to millimeter cubic size are considered to present brain function in a relatively macroscopic scale. Although there is no consensus to treat voxel‐wise fMRI signals as populational coding of cellular‐specific neuronal activity, the emerging optogenetic tools in combination with fMRI have opened a new avenue to decipher the cellular component contribution to the fMRI signal in animal models.^[^ ^8^, ^9^, ^10^ ^]^ However, to provide an insightful interpretation of the fMRI signal, the contribution of vascular components to the fMRI signal should be better elucidated, particularly with the evolved MR technology to improve the spatiotemporal resolution of fMRI brain mapping.

With state‐of‐the‐art high‐field MR technology, human brain fMRI has acquired functional maps with a spatial resolution of 300–500 µm voxels,^[^ ^11^, ^12^, ^13^, ^14^, ^15^ ^]^ which could well separate the pial vessels with near hundred‐micron diameters. In contrast to human brain mapping, high‐field rodent fMRI has enabled 2D slice image acquisition with 100 × 100 µm^2^ in‐plane resolution across the cortex.^[^ ^16^ ^]^ Because the rodent brain has a smooth surface with micro‐vessels radially distributed throughout the cortex, the vascular partial volume contribution to the high‐resolution fMRI signals of rodent brains is not negligible.^[^ ^17^ ^]^ Given the T2^*^ extravascular amplification effect, “single‐vessel” fMRI enables the detection of arteriole‐dominated cerebral blood volume (CBV) and venule‐dominated blood‐oxygen‐level‐dependent (BOLD) fMRI signals from intracortical vessel voxels.^[^ ^9^, ^16^, ^18^, ^19^ ^]^ A recent study has also detected vessel‐specific functional cerebral blood flow (CBF) velocity changes with high‐resolution phase‐contrast MRI.^[^ ^20^ ^]^ However, the increased spatial and temporal resolution of single‐vessel fMRI acquisition leads to an inevitable signal‐to‐noise ratio (SNR) loss. Previous single‐vessel fMRI methods apply reshuffled k‐t space fast low angle shot (FLASH) or balanced steady‐state free precession (bSSFP) sequences with focal field‐of‐view (FOV) to ensure sufficient SNR while maintaining the high spatiotemporal resolution.^[^ ^16^, ^21^, ^22^ ^]^ These methods also acquire single‐vessel images with less distorted images than the conventional echo‐planar imaging (EPI) method, but it remains challenging to push the resolution higher given the interdependent spatial, temporal resolution, and SNR.

To achieve the finest spatial scale for single‐vessel fMRI, we implement the radial encoding MRI scheme to measure the individual vessels penetrating the rat somatosensory cortex. In contrast to cartesian acquisition schemes, radial encoding offers continuous updating of the center of the k‐space and pushes the 50 × 50 µm^2^ spatial resolution with a 1 to 2 Hz sampling rate by defining the arbitrary number of projections in the azimuthal direction. Besides detecting refined hemodynamic maps of intracortical vessels, the radial encoding based single‐vessel fMRI offers the opportunity to distinguish the intravascular and extravascular effects from the cortical vessels.

## 2 Results

We implemented high‐resolution radial encoding based single‐vessel fMRI acquisition for in vivo hemodynamic measurement of individual penetrating arterioles and venules of anesthetized rats with a 14 T MR scanner. To better align the 2D radial encoding slice (FOV 9.6 × 9.6 mm^2^) along the cortical region of interest (Figure S1A, Supporting Information), we have adjusted the animal holder with a turning ring to allow one more degree of freedom to adjust the head rotation along the z‐axis of the MRI scanner.

### 2.1 Varied FOV Acquisition for Single‐Vessel Radial Encoding fMRI

**Figure 1:**
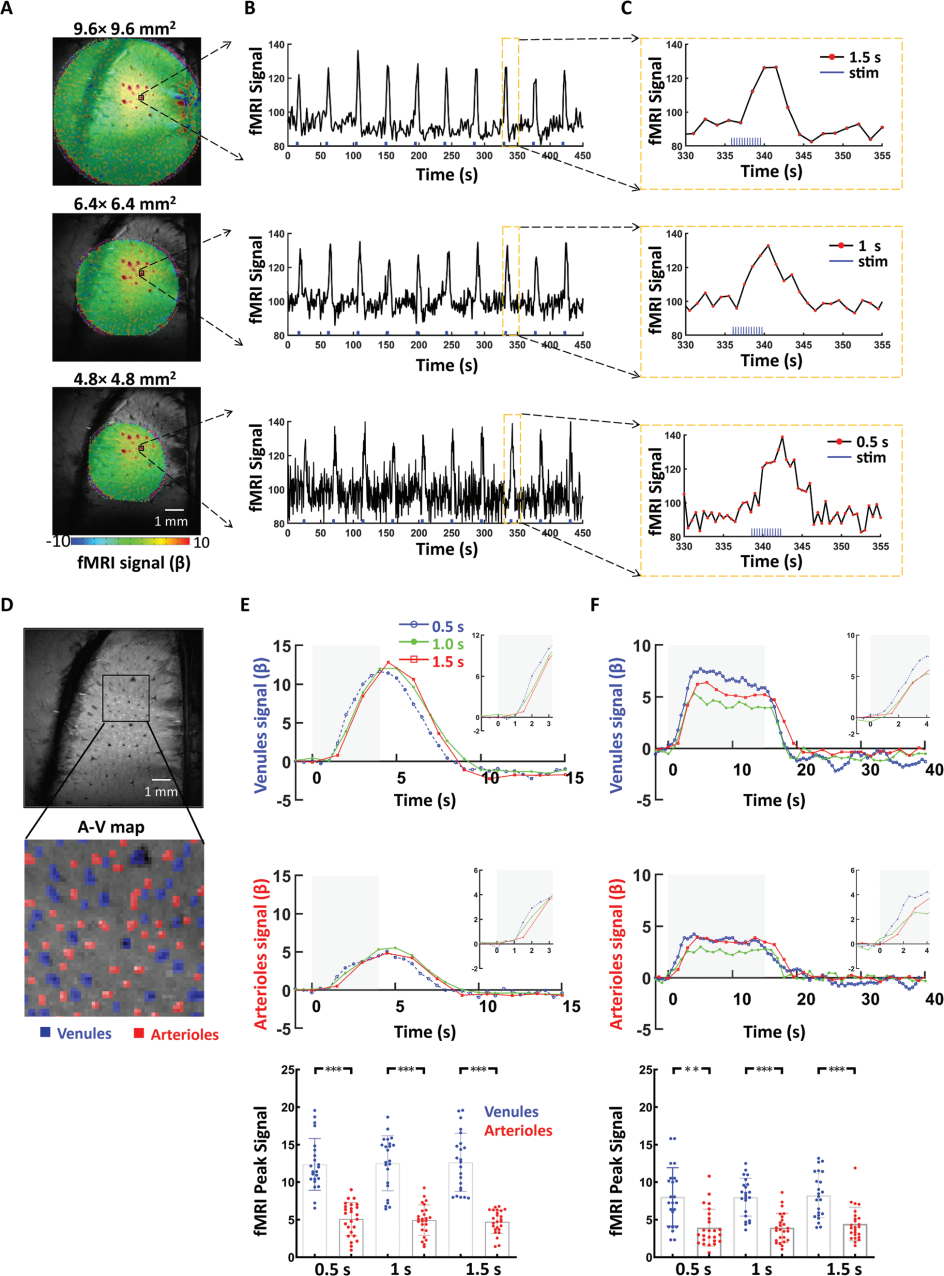
The single‐vessel radial encoding fMRI acquisition to achieve an arbitrary FOV. A) The FOV was reduced from 9.6 × 9.6 mm^2^ to 6.4 × 6.4 mm^2^ and 4.8 × 4.8 mm^2^ while covering the main responsive somatosensory cortex (FP‐S1) cortex with the reduced number of projections. B) The BOLD fMRI signal from a single venule in each FOV acquisition. C) The zoom‐in view of the plot in B (slice repetition rates of 0.5, 1, and 1.5 s) D) The vessel‐dominated BOLD fMRI A‐V map from the 2D MGE acquisition. The arteriole voxels were marked as red ROIs, and the venule voxels as blue ROIs. E) The averaged venules and arterioles of the evoked BOLD fMRI for different FOV acquisitions with sampling rates (0.5, 1, and 1.5 s). The block‐design paradigm for the forepaw stimulation train (330 µs, 1.5 mA) was delivered at 3 Hz for 4 s (E) and 3 Hz for 15 s F). The stimulation period is shown as a light‐gray shaded area. The zoomed views of the onset are outlined in each upper panel. The analysis of peak fMRI amplitude signals from individual venule (blue) and arteriole (red) voxels revealed markedly higher BOLD signals in venules compared to arterioles across different stimulation experiments, including durations of 4 s at 3Hz (E) and 15 s at 3 Hz (F) (^**^ *p* = 0.001, ^***^ *p* < 0.0001; unpaired *t*‐test; venule voxels, 23; arteriole voxels, 25). The error bars of this figure represent the mean ± standard deviation.

Since radial encoding applies the frequency‐encoding projection in a radial scheme, the aliasing effect of phase‐encoded direction will not affect the image acquisition. We first acquired the single‐vessel radial encoding fMRI images with three FOVs: 9.6 × 9.6, 6.4 × 6.4, and 4.8 × 4.8 mm^2^. **Figure** **1**A shows the overlaid BOLD fMRI maps on the multi‐gradient echo (MGE)‐based anatomical arteriole‐venule (A‐V) map, highlighting the activated venule voxels from the primary forepaw somatosensory cortex (FP‐S1) of anesthetized rats. As previously reported,^[^ ^9^, ^23^ ^]^ we applied an MGE sequence to acquire the A‐V map (Figure S1, Supporting Information), where the venule voxels appear as dark dots due to fast T2^*^ decay, while the arteriole voxels remain bright dots due to the in‐flow effect. The peak BOLD signals were mainly located on venule voxels, indicating venule‐dominated BOLD responses. Under the block‐design paradigm (stimulation on/off epochs), robust positive BOLD fMRI signals can be measured from individual venules with varied FOV. The reduction in FOV not only preserved the active cortical region but also allowed to decrease the slice repetition time from 1.5 to 0.5 s. The averaged time course of voxels from a single venule exhibited a robust response, and the overall contrast‐to‐noise ratio was high enough to detect single venule‐specific BOLD responses in the faster sampling scheme (Figure 1B,C). Figure 1D shows an example of the A‐V map with highlighted venule and arteriole ROIs. The BOLD fMRI signals of venule ROIs were significantly higher than those of arteriole ROIs, which were detected at 4 and 15 s duration stimulation paradigms with different slice repetition time (0.5, 1, and 1.5 s) (Figures 1E,F; S2, Supporting Information). These findings indicate the potential advantages of using smaller FOVs to detect vessel‐specific hemodynamic responses with a faster sampling rate using single‐vessel radial encoding fMRI.

### 2.2 Varied Spatial Resolution for Single‐Vessel Radial Encoding fMRI

**Figure 2:**
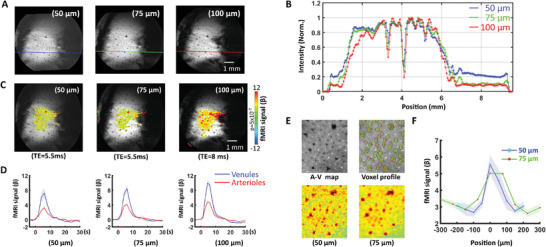
High‐resolution single‐vessel radial encoding fMRI recording acquisition. A) The different spatial resolutions achieved with single‐vessel radial encoding fMRI (in all cases, FOV = 9.6 × 9.6 mm^2^). B) The corresponding line intensity profile across the vessels from each resolution acquisition (line position indicated in panel (A). A clear point‐spreading function of the individual vessel can be indemnified from the 50 µm resolution fMRI. The intensity is normalized to the respective maximum. C) The BOLD fMRI maps at various resolutions were superimposed within the same 9.6 × 9.6 mm^2^ FOV. The TE was 5.5 ms for 50 and 75 µm resolution, and 8 ms for 100 µm resolution acquisition. D) The BOLD fMRI signal originating from venules and arterioles in the active cortex region of different resolution acquisition (*n* = 4 rats, mean ± SD). E) The parenchyma voxel profile extraction (along the pink line) for voxels within the individual venules mask (one venule voxel size was selected) and the corresponding BOLD fMRI distribution at 50 and 75 µm resolutions under the same TE (5.5 ms). F) The averaged venules‐specific BOLD fMRI profile patterns at 50 and 75 µm resolution from E (*n* = 4 rats, mean ± SEM).

By increasing the azimuthal projections at the same FOV (9.6 × 9.6 mm^2^) from 76 to 100 and to 150 projections, single‐vessel radial encoding fMRI enabled to increase the in‐plane resolution of hemodynamic mapping from100 × 100 µm^2^ to 75 × 75 µm^2^ and 50 × 50 µm^2^, respectively. When comparing the anatomical characterization of vessel voxels from different raw images, the most distinct and well‐defined spreading function of individual venules could be identified in radial encoding images of 50 × 50 µm^2^ (**Figure** **2**A,B). The BOLD functional maps were also obtained by single‐vessel radial encoding fMRI with different spatial resolutions, demonstrating much more refined hemodynamic maps overlaying on the A‐V maps (Figure 2C). We further analyzed the hemodynamic characteristics of vessel‐specific BOLD fMRI signals at different spatial resolutions. Using the A‐V map‐based vessel ROIs, the venule‐specific BOLD responses of 50 µm resolution represent relatively lower amplitude than the 100 µm resolution due to altered partial volume extravascular effect from different voxel sizes (Figure 2D). Meanwhile, the BOLD signal from individual venules ROIs acquired at 50 µm resolution shows sharper profile distribution and higher peak responses than 75 µm under the same TE acquisition (Figure 2E,F), indicating that more refined vessel‐specific BOLD responses are detected with the version of the sequence employing higher spatial resolution.

**Figure 3:**
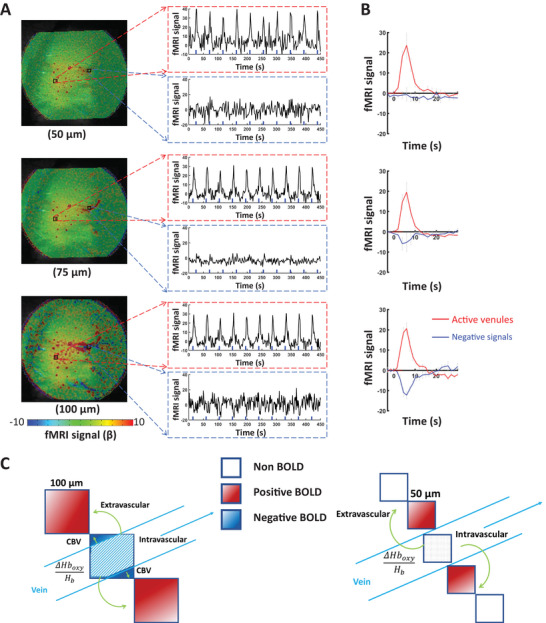
Intravascular effect of draining veins of high‐resolution single‐vessel radial encoding fMRI. A) The distributions of BOLD fMRI maps in‐plane resolution range from 50 to 100 µm (FOV 9.6 × 9.6 mm^2^). The positive fMRI signals from single venules and negative signals surrounding the pial veins. The position and voxel signal time courses in active venules and voxels surrounding the draining veins (Figures S3 and S4, Supporting Information). B) The average positive and negative BOLD signals from different animals (*n* = 4 rats, mean ± SEM). C) The schematic diagram illustrates the impact on intravascular and extravascular effects caused by changes in voxel size.

Moreover, the single‐vessel radial encoding fMRI maps obtained at different resolutions enabled the direct characterization of distinct intravascular and extravascular effects from large pial vessels. Depending on the slice localization, the low spatial resolution scheme (100 × 100 µm^2^) showed not only positive BOLD signals in venule voxels but also negative BOLD signals in voxels covering the pial surface vein (**Figure** **3**A; Figure S3, Supporting Information). The negative BOLD signal can be caused by the passive venule dilation (i.e. reduced T2^*^ signal due to the CBV effect),^[^ ^24^, ^25^, ^26^ ^]^ which will primarily affect voxels with 100 × 100 µm^2^ in‐plane size, covering both blood and parenchymal tissue. The surrounding positive BOLD signals are caused by the typical extravascular effect of oxy/deoxy‐hemoglobin ratio increase. However, the negative signal disappeared at higher spatial resolution radial encoding fMRI mapping with positive BOLD signals surrounding the pial veins (Figure 3B; Figure S4, Supporting Information). This is possibly due to the fact that a smaller voxel size enables the detection of the intravascular blood signals that are diminished at high magnetic field^[^ ^27^ ^]^ without the negative CBV effect (Figure 3C). This radial encoding based high‐resolution fMRI has presented highly refined vessel‐specific hemodynamic responses of rat brains, enabling the dissection of vessel‐specific contributions to fMRI signals.

### 2.3 Comparison of Single‐Vessel Radial Encoding and bSSFP fMRI

**Figure 4:**
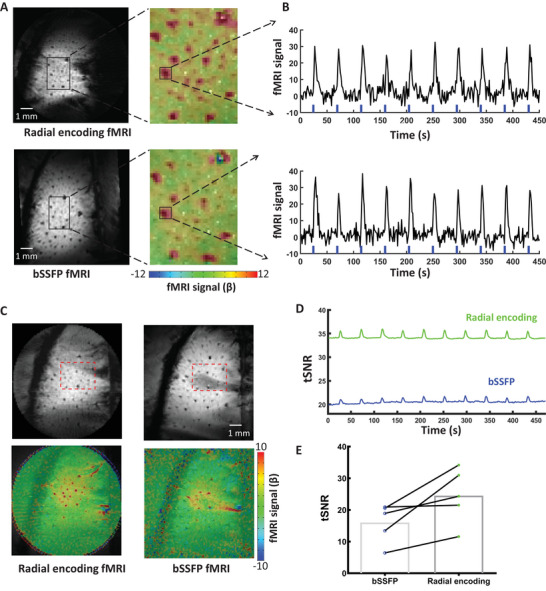
Comparison of single‐vessel radial encoding fMRI and bSSFP fMRI. A) The 100 µm in‐plane resolution single‐vessel radial encoding fMRI and bSSFP fMRI image and BOLD maps (FOV 9.6 × 9.6 mm^2^, 40% transparency fMRI map). B) The positive fMRI signals from single venules from single‐vessel radial encoding fMRI and bSSFP fMRI. C) The representative images and fMRI maps of single‐vessel radial encoding and bSSFP acquisition. In bSSFP acquisition, banding artifacts are evident in the active cortex region of some animals, leading to a reduction in fMRI signals. D) The representative time course of temporal Signal‐to‐Noise Ratio (tSNR) of single‐vessel radial encoding and bSSFP fMRI acquisition. The rectangular mask for the active region of interest (ROI) is displayed in panel C. E) A paired comparison of the mean tSNR values for single‐vessel bSSFP and radial encoding fMRI from the same animal acquisition (*n* = 5 rats, paired *t*‐test, *p* < 0.02).

We also conducted a comparison between single‐vessel radial encoding and bSSFP fMRI focusing on the 100 × 100 µm^2^ in‐plane resolution in the same animals. Both methods revealed highly robust positive BOLD fMRI signals located at the venule voxels (**Figure** **4**A), demonstrating the compelling consistency of the single‐vessel venule‐dominated BOLD detection (Figure 4B). Meanwhile, it is important to note the presence of potential bSSFP banding artifacts in the cortex due to field inhomogeneities (Figure 4C). Supplementary movie 1 presents representative evidence of these banding artifacts and the pattern of artifacts that evolved during the acquisition due to the altered gradient/shimming coil temperature. Also, the radial encoding fMRI dataset showed significantly higher tSNR than bSSFP datasets (Figure 4C–E), indicating better detectability of the single‐vessel radial encoding scheme for measuring vessel‐specific hemodynamic responses.

## 3 Experimental Section

### 3.1 Animal Preparation

All animal surgical and experimental procedures were in full compliance with the guide for the care and use of laboratory animals and approved by the Massachusetts General Hospital Institutional Animal Care and Use Committee. Male Sprague Dawley rats (≈250g) were intubated with a mechanical ventilator (SAR‐830, CWE, USA) and anesthetized with 2% isoflurane. Blood pressure monitoring and anesthetics infusion took place via femoral artery and vein catheterization. Isoflurane was discontinued after i.v. bolus injection of α‐chloralose (Sigma–Aldrich, 80mg kg^−1^) through the femoral vein. During MR acquisition, the α ‐chloralose (26.5mg kg^−1^ h^−1^) mixed with pancuronium (Zemuron, 2 mg kg^−1^ h^−1^) was infused to immobilize the rats. Also, a heating pad was used to maintain the rectal temperature of rats at ≈37 °C for the duration of the experiment. All relevant physiological parameters were constantly monitored during scanning: heart rate, arterial blood pressure, pressure of the tidal ventilation (Biopac MP 160, Biopac Systems, USA), and end‐tidal CO_2_ (Capnometer, Novametrix).

### 3.2 MRI Acquisition

MRI images were acquired using a 14 T, 26 cm horizontal bore magnet (Magnex Scientific) interfaced through the Bruker Advance III console (Bruker Corporation). A 12 cm magnet gradient set was equipped with a strength of 100 G cm^−1^ and a 150 µs rise time (Resonance Research Inc.) in the scanner. The stimulation paradigm was triggered directly through the MRI scanner and was controlled by Master‐9 (A.M.P.I system, Jerusalem, Israel). The triggering pulses from the MRI scanner were also recorded by a Biopac system (MP160, Biopac Systems, USA). To map the sensory‐evoked single‐vessel fMRI, a pair of stimulation electrodes were placed on the forepaw to deliver trains of 1.5 mA pulses (300 µs) at 3Hz for 4s or at 3Hz for 15s in each fMRI epoch. A 6 mm transceiver coil was constructed and attached to the rat skull covering the somatosensory cortex.

### 3.3 Single‐Vessel Multi‐Gradient Echo (MGE) Imaging

To acquire the anatomical arteriole‐venule (A‐V) map, a 2D multiple gradient echo (MGE) sequence was implemented with the following parameters: TE: 2.5, 5, 7.5, 10, 12.5, 15 ms; TR: 50 ms; slice thickness: 500 µm; flip angle: 55°; matrix: 192 × 192; in‐plane resolution: 50 × 50 µm^2^. The single vessel map was acquired by averaging the MGE images acquired from the second echo to the fourth echo.

### 3.4 Single‐Vessel Radial Encoding BOLD fMRI

Radial encoding acquisition with varying parameters was employed to investigate spatial and temporal freedom in fMRI. For temporal freedom acquisition, a slice thickness of 500 µm, an undersampling factor of 2, and flip angle of 15° were utilized. The parameters of single‐vessel radial encoding BOLD fMRI acquisition are summarized in **Table** **1**. The following parameters were used for a FOV of 9.6 × 9.6 mm^2^ acquisition: TE of 8 ms; TR of 19.738 ms; 76 projections; matrix size of 96 × 96, 1.5 s scan time per slice. For a reduced FOV of 6.4 × 6.4 mm^2^: TE of 8 ms; TR of 20 ms; 50 projections, matrix size of 64 × 64, 1 s scan time per slice. To achieve a sampling rate of 2 Hz and cover the responsive cortical region with a FOV of 4.8 × 4.8 mm^2^, TE of 8 ms and TR of 13.159 ms were applied; 38 projections; matrix size of 48 × 48. For FOV 9.6 × 9.6 mm^2^, the block design comprised 25 pre‐stimulation scans, 1 scan for stimulation trigger, and 29 post‐stimulation scans. Each trial consisted of 20 epochs with a total scan duration of 15 min and 37 s. Similarly, for FOV 6.4 × 6.4 mm^2^, the block design was 25 pre‐stimulation scans, 1 scan for stimulation trigger, and 44 post‐stimulation scans with 20 epochs for each trial with a total scan duration of 15 min and 22 s. For FOV 4.8 × 4.8 mm^2^, the block design was 50 pre‐stimulation scans, 1 scan for stimulation trigger, and 89 post‐stimulation scans with 20 epochs for each trial.

**Table 1:** Summary of parameters for single‐vessel radial encoding BOLD fMRI.

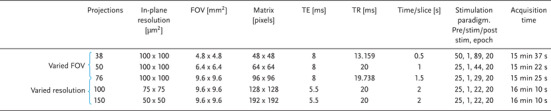

To investigate in‐plane spatial resolutions of 50, 75, and 100 µm, single‐vessel fMRI images with varying numbers of projections and matrix sizes were recorded. Specifically, for 50 µm resolution, a matrix size of 192 × 192, 150 projections, TR of 20 ms, TE of 5.5 ms, and 2 s scan time per slice was used. For the 75 µm resolution acquisitions, the matrix size was 128 × 128, and 100 projections were used, TR of 20 ms, TE of 5.5 ms, and 2 s scan time per slice. For the 100 µm resolution acquisitions, the matrix size was 96 × 96, and 76 projections were used. These settings allowed for the assess the impact of spatial and temporal freedom acquisition in radial encoding fMRI. For the in‐plane resolution of 50 and 75 µm acquisition, the block design was 25 pre‐stimulation scans, 1 scan for the stimulation trigger, and 22 post‐stimulation scans with 20 epochs for each trial with a total scan duration of 16 min and 10 s.

### 3.5 Balanced Steady‐State Free Precession (bSSFP) Single‐Vessel BOLD fMRI

For real‐time bSSFP fMRI acquisition, the following parameters were used: TE of 7.8 ms; TR of 15.625 ms; flip angle of 15˚; FOV of 9.6 × 9.6 mm^2^; matrix size of 96 × 96; one slice repetition time of 1.5 s. The fMRI block design consisted of 25 pre‐stimulation scans, 1 scan for stimulation trigger, and 44 post‐stimulation scans with 20 epochs. The total acquisition duration of each trial was 15 min and 37 s. The geometry of bSSFP was set to the same as the 100 µm radial encoding MRI.

### 3.6 Data and Statistical Analysis

All data preprocessing and analysis were performed using the software package Analysis of Functional NeuroImages (AFNI) (NIH, Bethesda, USA) and MATLAB (MathWorks, Natick, USA). All relevant fMRI analysis source codes can be downloaded from https://afni.nimh.nih.gov/. A detailed data analysis description of the processing can be found in a previous study.^[^ ^9^, ^20^ ^]^ To register the evoked single‐vessel fMRI images with the 2D anatomical A‐V map, the tag‐based registration method was applied. The classification of individual vessel voxels was based on their signal intensity. Voxels with signal intensity higher than the mean signal intensity plus three times the standard deviation were color‐coded in red, representing arterioles. On the other hand, voxels with signal intensity lower than the mean signal intensity minus three times the standard deviation of local areas (in a 5 × 5 kernel) were color‐coded in blue, representing venules. Multiple trials of block‐design time courses were averaged for each animal. No additional smoothing step was applied. The 3dDeconvolve module developed in AFNI was used to map the hemodynamic response based on the “block function”. The hemodynamic model is defined as follows:

(1) $$h\;\left(t\right)=\int_{0}^{\min\left(t,L\right)}s^{4}\;e^{-s}/\left[4^{4}e^{-4}\right]\;\mathrm{ds}$$

*L* is the duration of the response. BLOCK (L,1) is a convolution of a square wave of duration L with gamma variate function = s^4^e^−s^ /4^4^e^−4^, making a peak amplitude of block response 1. The fMRI β‐value was calculated to measure the amplitude of the fMRI responses at each slice frame. The peak fMRI signals were extracted from individual venule and arteriole voxels. The line intensity profile for high‐resolution single‐vessel fMRI was normalized to the respective maximum. The tSNR was computed from the mean value of 30 × 20 voxels in the center of the activation region across all the fMRI time points, divided by the temporal standard deviation of the voxels.

For the group analysis, a Student's *t*‐test was performed. The error bars in all figures represent mean ± standard error of the mean (SEM) unless otherwise indicated. The threshold for statistically significant was set at *p* < 0.05.

## 4 Discussion

High spatial resolution fMRI in animal models has evolved to identify individual penetrating microvessels with distinct vessel‐specific hemodynamic responses. This single‐vessel fMRI approach allows non‐invasive detection of large‐scale hemodynamic signals from large animals without spectral‐specific signal attenuation through the skull, for example ultrasound or optoacoustic imaging.^[^ ^28^, ^29^, ^30^ ^]^ The present study has implemented the radial encoding scheme to further push the spatial resolution of fMRI to 50 µm in‐plane with sub‐second TR, aiming to dissect the vascular contributions to fMRI signal.

### 4.1 Advantage of the Radial Encoding Scheme for Ultra‐High‐Resolution fMRI

With the conventional cartesian trajectory k‐space acquisition, faster sampling can be achieved by using a reduced matrix and phase encoding steps with a smaller in‐plane FOV,^[^ ^31^ ^]^ but the aliasing effect can lead to fold‐over artifacts. Anti‐aliasing encoding schemes can be used but this often comes at the cost of decreased temporal resolution. Although the echo planar imaging (EPI) method enables fast sampling through the echo trains with a prioritized wide range of brain coverage, the detected hemodynamic response can suffer from distortion due to the embedded T2^*^ effect during acquisition. To enable the acquisition of small FOV, the radial encoding sampling scheme has been implemented.^[^ ^32^, ^33^ ^]^ This radial encoding method presents numerous advantageous features for ultra‐high resolution fMRI brain mapping. To mitigate the aliasing artifacts, radial encoding implemented a radial frequency encoding scheme with less fold‐over issues due to the phase‐encoding scheme. The undersampling of radial encoding with a low number of projections can result in streak artifacts.^[^ ^34^, ^35^ ^]^ In our study, we employed 38 radial projections for a small FOV (FOV 4.8 × 4.8 mm^2^, matrix size 48 × 48, in‐plane resolution 100 × 100 µm^2^) at the undersampling factor of two that effectively mapped the BOLD fMRI signals from the somatosensory cortex of rat brains without encountering obvious streaking artifacts. Also, radial encoding enables repeated sampling in the k‐space center to ensure an overall uniform contrast. By reducing projections and matrix size in the azimuthal direction, image acquisition can be accelerated to only focus on a smaller FOV (e.g. 4.8 × 4.8 mm^2^) with high spatial resolution, enabling single‐vessel fMRI with a faster sampling rate. This approach is advantageous because it focuses on the region of interest where the highest signal energy is frequently concentrated, allowing for higher temporal resolution. A constant azimuthal profile spacing (111.246°) based on the golden ratio can further be applied to optimize image reconstruction from a more uniform profile distribution in radial MRI.^[^ ^36^ ^]^ The golden angle radial scheme can facilitate dynamic imaging through continuous data acquisition and retrospective reconstruction of image series. This approach can be effectively integrated with respiratory or cardiac motion, along with compressed sensing reconstruction techniques.^[^ ^33^, ^37^ ^]^ By narrowing the ROI and capturing the most relevant signal information, the single‐vessel radial encoding method enhances the accuracy and fidelity of the hemodynamic response measurement. Additionally, the multiple coils of the undersampled radial trajectories acquisitions and nonlinear algorithm reconstruction methods can further suppress streak artifact.^[^ ^38^, ^39^ ^]^

### 4.2 Comparison to the Previously Developed Single‐Vessel fMRI Methods

The usage of reshuffled k‐t space FLASH for single‐vessel fMRI has demonstrated remarkable capabilities in achieving high spatiotemporal resolution,^[^ ^9^ ^]^ however, it is not a real‐time fMRI acquisition. Recently, a new approach called direct imaging of neuronal activity (DIANA),^[^ ^40^ ^]^ based on 2D shuffled line scanning has been proposed.^[^ ^9^, ^41^, ^42^ ^]^ There has been an ongoing debate regarding the reproducibility of the DIANA method.^[^ ^22^, ^23^, ^24^ ^]^ One ongoing concern is the temporal aliasing issue with this reshuffled k‐t space trajectory,^[^ ^43^, ^44^, ^45^ ^]^ which could make the detected ultra‐fast dynamic signals confounded by both physiological and non‐physiological noises.

An alternative method, the bSSFP method, has gained recognition for its ability to offer real‐time monitoring of hemodynamic signals and superior SNR efficiency compared to other pulse sequences.^[^ ^21^, ^22^ ^]^ Previously, He et al. have applied the bSSFP for single‐vessel hemodynamic signal detection with an in‐plane resolution of 100 × 100 µm^2^.^[^ ^16^, ^43^ ^]^ Typically, SSFP applications focus on the pass‐band region, where a high SNR per unit time can be attained while being relatively less affected by precise off‐resonance frequencies. To mitigate this off‐resonance banding artifact, careful shimming adjustments need to be periodically applied during the experiment. As long as effective shimming is employed to confine the frequency range within the pass‐band in the anatomical region of interest, the resulting image will exhibit uniform signal and contrast. It is worth noting that when using high spatial resolution from focal FOV with short TR/TE, the demanded strong gradient in the high‐duty cycle bSSFP sequence leads to localized temperature increases in the gradient which alter the shimming outcomes during scanning (Figure 4A; Movie S1, Supporting Information). This challenging issue is a main course of the banding artifacts of the bSSFP‐based single‐vessel fMRI.

The radial encoding scheme allows the reconstruction of the fMRI images based on the number the azimuthal projections. This method provides the flexibility to alter the FOV, as well as the corresponding spatial and temporal resolution. One challenging issue is to position the brain region of interest to the geometrical center of the magnet (or the gradient). We have produced an animal holder to ease the animal head positioning inside the MRI scanner (Figure S1, Supporting Information), allowing the setup of geometrical orientation for radial encoding fMRI to target the regions of interest in rat brains.

### 4.3 The Focus Shifted from Functional MRI to Hemodynamic MRI

Numerous efforts have been made to improve the spatial specificity of fMRI by eliminating the draining vein‐mediated mislocalization of functional maps.^[^ ^5^, ^27^, ^46^, ^47^, ^48^, ^49^, ^50^, ^51^ ^]^ These studies aim to present the brain function with fMRI by emphasizing the hemodynamic responses from intracortical voxels enriched with arterioles and capillaries, which have a closer proximity to the neuronal sources.^[^ ^23^, ^52^, ^53^ ^]^ One fundamental difference between the high‐resolution single‐vessel fMRI with the previous studies is that it targets the vascular origin of fMRI. Thus, in contrast to the “functional” term of fMRI, the single‐vessel fMRI approach focuses on the hemodynamic features, which makes it more appropriate to be called a single‐vessel “hemodynamic” (h)MRI.

Different from the advanced optical imaging, optoacoustic, and functional ultrasound imaging schemes, the unique non‐invasive and global mapping features of single‐vessel fMRI ensures the detection of less disturbed neurovascular coupling, as well as being less dependent on the vascular flow orientation than the Doppler effect. Nevertheless, given the shifted focus on vascular specificity, the challenge of single‐vessel fMRI is to detect more refined micro‐vessels with high spatiotemporal resolution. Single‐vessel radial encoding fMRI proposed in this study is an ongoing effort to map the penetrating micro‐vessels from rodent brains. In particular, the 50 × 50 µm^2^ in‐plane resolution single‐vessel maps enable the well‐characterized BOLD fMRI signals from refined arteriole and venule voxels, attributing more vessel‐specific hemodynamic features.

Besides the intracortical micro‐vessel fMRI mapping, our work also exemplified the differentiation of fMRI signals from vessel and peri‐vessel voxels near pial vessels. With the typical spatial resolution at the submillimeter scale, the intravascular contribution to the fMRI signal at high field is considered to be negligible given the low partial volume fraction of the cerebrovasculature (<4%) and the fast T2^*^ decay of blood signals, in particular, for the deoxygenated blood of venules/veins.^[^ ^27^ ^]^ Here, we achieved 50 × 50 µm^2^ in‐plane resolution from the 2D slice covering the cortical surface draining veins. At such a high resolution, it offers an opportunity to identify the finer vascular contribution to the fMRI signals based on the localization of voxels: vessel voxels or peri‐vessel voxels. As shown in Figure 3, when the voxel size was small enough (e.g., 50 × 50 µm^2^ in‐plane resolution) to be directly positioned within the draining vein, no fMRI signal was detected in the vessel voxels. This is mainly due to the fast diminished T2^*^ signals from the blood of draining veins, that is the intravascular effect on fMRI signals. In contrast, when the voxel was slightly bigger (e.g., 100 × 100 µm^2^ in‐plane resolution) to cover the draining vein and adjacent parenchyma, we observed a negative fMRI signal due to the passive dilation of the draining vein,^[^ ^16^, ^24^, ^49^ ^]^ that is the increased partial volume contribution of blood to the given voxels. Meanwhile, the voxels near the vein (peri‐vessel voxels) sense the extravascular effects based on the improved field homogeneity upon the activity‐coupled increase of oxygenated blood flow into the draining veins. Thus, we have described altered fMRI signals from vessel and peri‐vessel voxels depending on the distinct contribution of intravascular and extravascular effects. It should be noted that hyperemia‐driven venous dilation upon neuronal activation has not been reliably detected with optical imaging. This raises the alternative explanation: the negative fMRI signals may be detected near the diving arteries instead of veins. Future studies will be focused on differentiating the intravascular and extravascular effects from pial arteries and veins using the ultra‐high resolution radial encoding fMRI method.

## 5 Summary

The single‐vessel radial encoding fMRI method involves aligning the k‐space line radially to increase temporal resolution when mapping focal cortical regions with reduced artifacts. The ability to assign different projection numbers provides unique flexibility to develop more efficient and customizable data acquisition strategies, resulting in improved spatial resolution and localization specificity in fMRI studies. Moreover, this dynamic imaging approach demonstrated superior tSNR compared to bSSFP fMRI, making it particularly suitable for real‐time fMRI applications. This radial encoding scheme enables dynamic imaging using continuous data acquisition and retrospective reconstruction of image series, which can be combined with respiratory or cardiac motion and compressed sensing reconstruction techniques.^[^ ^33^, ^54^ ^]^ This combination further benefits real‐time single‐vessel BOLD fMRI, CBV, and cerebral blood flow studies, making it a valuable tool for advanced brain functional mapping with high‐field MRI scanners.

## Conflict of Interest

The authors declare no conflict of interest.

## Supporting information

Supporting Information

Supplemental Movie 1

## Data Availability

The data that support the findings of this study are available from the corresponding author upon reasonable request.
