# Supplementary material for: High Spatiotemporal Resolution Radial Encoding Single‐Vessel fMRI
Source: Adv Sci (Weinh). 2024 Apr 30;11(26):2309218. doi: 10.1002/advs.202309218 (PMC11234406; doi:10.1002/advs.202309218)
Supplement: Supplementary file 1 — Supporting Information [file ADVS-11-2309218-s001.pdf]

ADVANCED SCIENCE
Open Access

Supporting Information

for *Adv. Sci.*, DOI 10.1002/advs.202309218

High Spatiotemporal Resolution Radial Encoding Single-Vessel fMRI

*Yuanyuan Jiang, Patricia Pais-Roldán, Rolf Pohmann and Xin Yu**

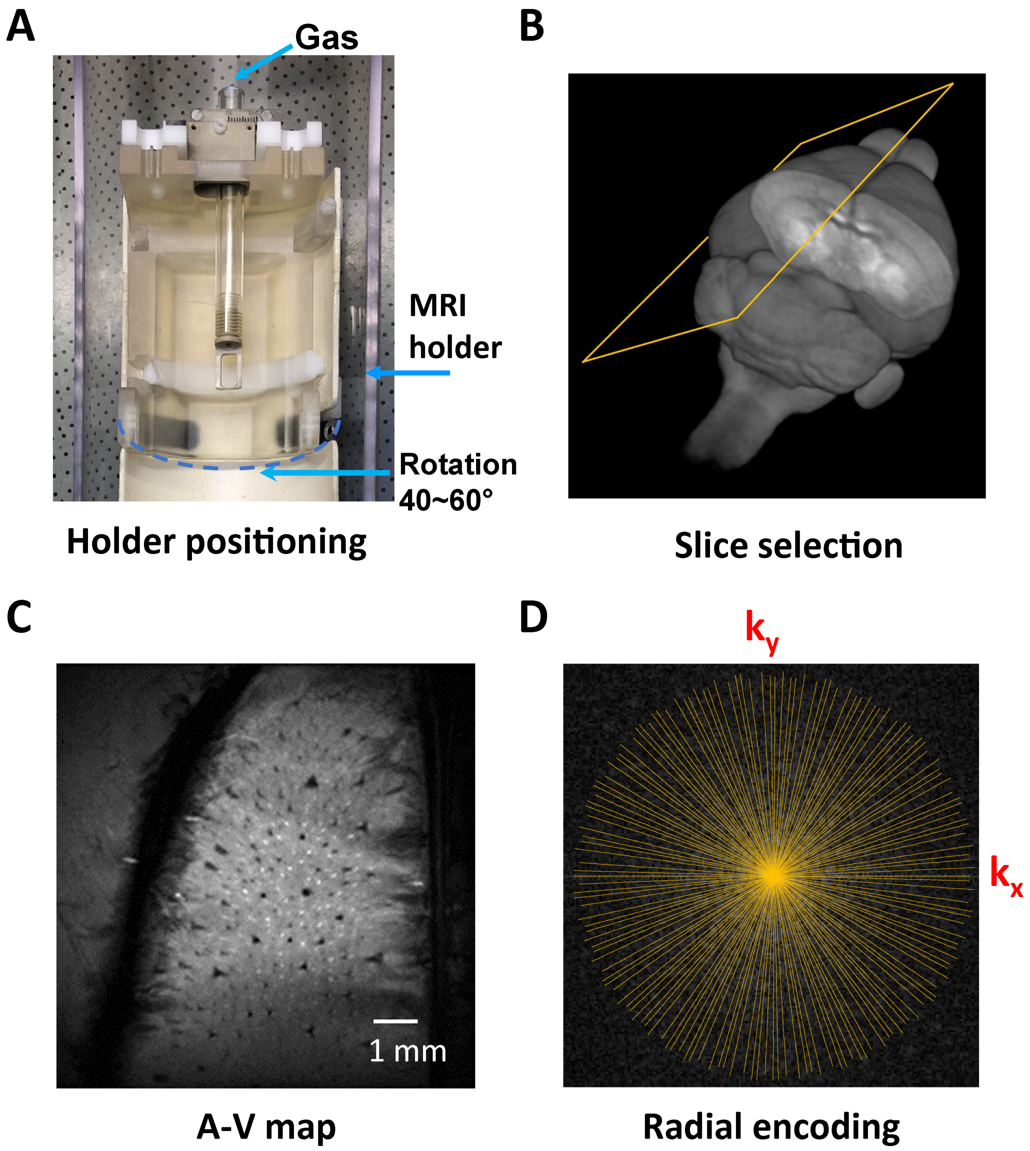

Fig. S1. The 2D golden angle radial encoding MRI approach for *in vivo* fMRI experiment. (**A)** The rotatable animal holder for animal head positioning freedom in 14T MRI scanner. (**B)** 2D slice MRI selection for single-vessel radial encoding fMRI mapping of somatosensory cortex. (**C**) A representative anatomical 2D A-V (arteriole–venule) map acquired by multi-gradient echo (MGE) sequences. (**D)** The radial encoding scheme allows single-vessel fMRI acquisition with an arbitrary number of profiles for the radial sampling of k-space.

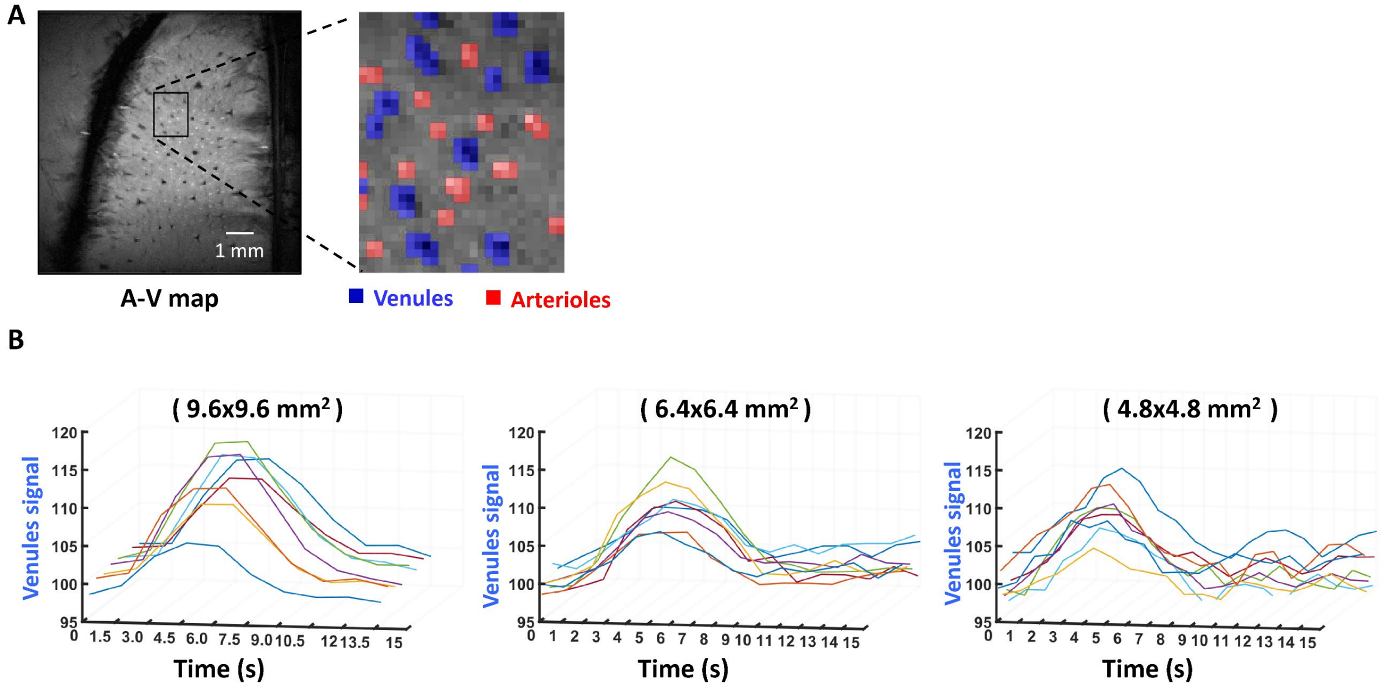

Fig. S2. The representative venule-specific time course for radial encoding single vessel fMRI. (**A**) The individual arteriole and venule voxels were extracted from the A-V map with different signal intensities (venule voxels, blue; arteriole voxels, red). (**B**) Individual venule voxel signal at different FOV acquisitions (9.6x9.6, 6.4x6.4, and 4.8x4.8 mm²) from one representative rat.

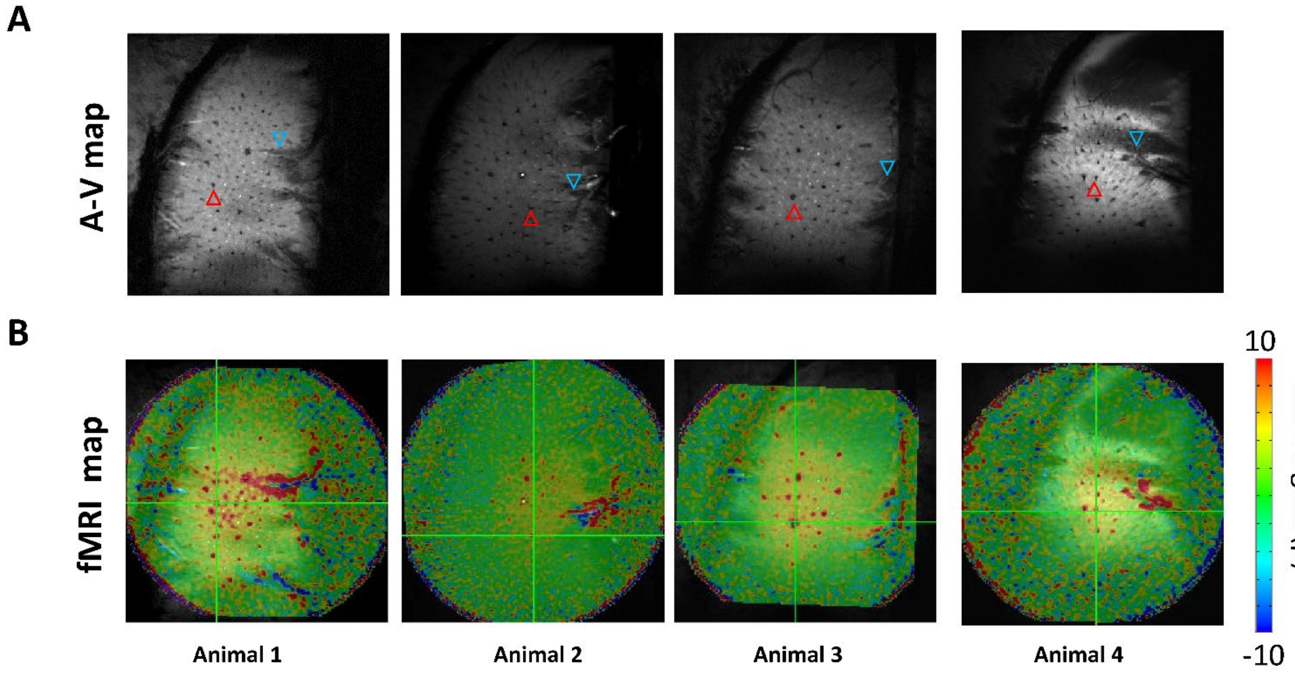

Fig. S3. The positive BOLD signal and negative BOLD signals can be detected surrounding the pial veins with 100x100 μm^2 resolution single-vessel fMRI. (**A**) 2-D slice A-V map of 4 representative animals. The red and blue triangle denotes the representative positive venules from the active cortex and negative BOLD signals surrounding the pial veins in the A-V map. (**B**) The corresponding fMRI map (100x100 μm^2 resolution) was overlaid on the corresponding A-V map (4 animals).

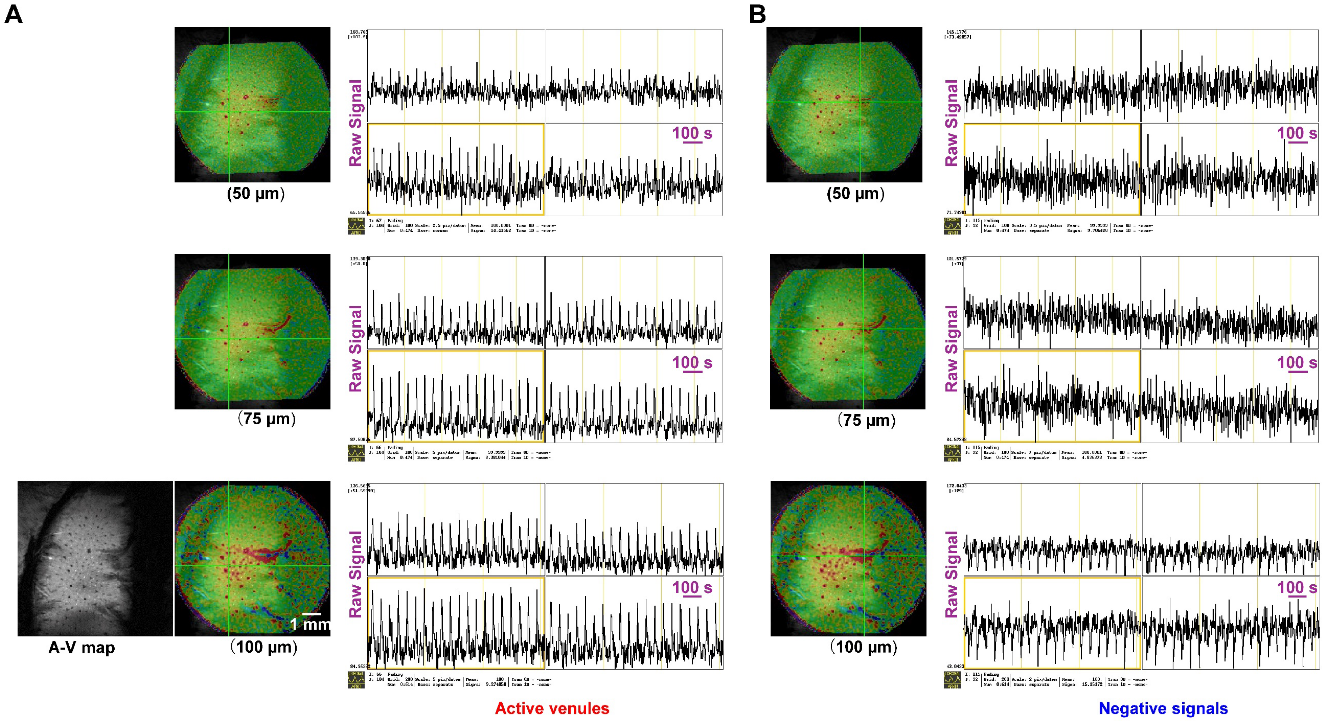

Fig. S4. The fMRI mapping and time course of positive BOLD and negative BOLD signals at different resolution radial encoding based single-vessel fMRI. (**A**) The representative positive BOLD signal from one venule voxel from the active somatosensory cortex from different spatial resolutions (50, 75, and 100 µm). (**B**) The negative BOLD signal surrounding the pial veins was reduced with a higher spatial resolution single-vessel radial encoding fMRI.

Movie S1.

This movie shows the signal variations in radial encoding based single-vessel and bSSFP fMRI at the same resolution. The positive BOLD fMRI time course of bSSFP fMRI (upper panel) and radial encoding single-vessel fMRI (lower panel) were extracted from the same vessel location. The video showcases representative patterns of banding artifacts evolving during the bSSFP acquisition, attributed to changes in gradient/shimming coil temperature.
